# Highly Resolved Phylogenetic Relationships within Order Acipenseriformes According to Novel Nuclear Markers

**DOI:** 10.3390/genes10010038

**Published:** 2019-01-10

**Authors:** Dehuai Luo, Yanping Li, Qingyuan Zhao, Lianpeng Zhao, Arne Ludwig, Zuogang Peng

**Affiliations:** 1 Key Laboratory of Freshwater Fish Reproduction and Development (Ministry of Education), Southwest University School of Life Sciences, Chongqing 400715, China; ldh2015@email.swu.edu.cn (D.L.); liyanping@genomics.cn (Y.L.); zqingyuanbiology@sina.com (Q.Z.); xndxsky_zlp@sina.com (L.Z.); 2 Shenzhen Key Lab of Marine Genomics, Guangdong Provincial Key Lab of Molecular Breeding in Marine Economic Animals, BGI Academy of Marine Sciences, BGI Marine, BGI, Shenzhen 518083, China; 3 Department of Evolutionary Genetics, Leibniz-Institute for Zoo and Wildlife Research, 10315 Berlin, Germany

**Keywords:** nuclear markers, phylogeny, Acipenseriformes, molecular clock

## Abstract

Order Acipenseriformes contains 27 extant species distributed across the northern hemisphere, including so-called “living fossil” species of garfish and sturgeons. Previous studies have focused on their mitochondrial genetics and have rarely used nuclear genetic data, leaving questions as to their phylogenetic relationships. This study aimed to utilize a bioinformatics approach to screen for candidate single-copy nuclear genes, using transcriptomic data from sturgeon species and genomic data from the spotted gar, *Lepisosteus oculatus*. We utilized nested polymerase chain reaction (PCR) and degenerate primers to identify nuclear protein-coding (NPC) gene markers to determine phylogenetic relationships among the Acipenseriformes. We identified 193 nuclear single-copy genes, selected from 1850 candidate genes with at least one exon larger than 700 bp. Forty-three of these genes were used for primer design and development of 30 NPC markers, which were sequenced for at least 14 Acipenseriformes species. Twenty-seven NPC markers were found completely in 16 species. Gene trees according to Bayesian inference (BI) and maximum likelihood (ML) were calculated based on the 30 NPC markers (20,946 bp total). Both gene and species trees produced very similar topologies. A molecular clock model estimated the divergence time between sturgeon and paddlefish at 204.1 Mya, approximately 10% later than previous estimates based on cytochrome b data (184.4 Mya). The successful development and application of NPC markers provides a new perspective and insight for the phylogenetic relationships of Acipenseriformes. Furthermore, the newly developed nuclear markers may be useful in further studies on the conservation, evolution, and genomic biology of this group.

## 1. Introduction

In 2010, the International Union for Conservation of Nature (IUCN) Red List of Threatened Species concluded that “Sturgeon more critically endangered than any other group of species”. Considering IUCN Red List data, twenty-seven species of sturgeon are listed with 63 percent as Critically Endangered—the Red List’s highest category of threat. Four species are now possibly extinct. Although progress was made in recovery of North American species during the last decade, the situation is dire for most Eurasian species. The latest update of the Red List assessed the status of 18 species of sturgeon from all over Europe and Asia and found that all were threatened (The IUCN Red List of Threatened Species). Within the Eurasian species, some are on the brink of extinction [1].

Mitochondrial genes have been used in molecular phylogenetic analyses and species identification since the 1990s. Their high copy number, easy amplification, and fast evolutionary rate [2,3] make them valuable markers addressing taxonomic issues in a wide range of species. However, there are important drawbacks to their use, such as their being inherited as a unit [3]. Therefore, all mitochondrial genes should be considered collectively as a single marker no matter how many genes are concatenated. Many empirical studies have suggested that the rate of mitochondrial evolution is fast, which limits the value of mitochondrial loci in deep-level phylogeny [4]. Nuclear molecular markers are the preferable choice for the phylogenetic and conservation genetic studies of ancient groups, and have been utilized in many species [5,6,7,8,9,10,11,12]. There are three main classes of molecular markers: nuclear protein coding loci exon-primed intron-crossing markers, and anonymous nuclear markersthat have been successfully developed in non-model organisms to address a wide range of questions in phylogenetics and phylogeography [13]. Nuclear protein coding loci are used most frequently for several reasons, including their straightforward handling, low likelihood of paralogs, high number potential loci, and large phylogenetic span [13]. Meanwhile, exons are generally conservative and evolve slowly, making them suitable for the study of deep-level phylogenetics [4,14].

The Acipenseriformes lineages represent some of the most primitive lineages of recent vertebrates [15], with many members of the group under threat [16]. Acipenseriformes represent the “living fossils” of aquatic life on earth and have a history of more than 200 million years [17]. Many species are polyploid [16,18,19], but no reference genome sequence data have been published to date depending on their huge genome sizes. This has led to a focus on mitochondrial genes to establish phylogenetic relationships, with nuclear marker data lacking [15]. However, the results of molecular and morphological studies are controversial, generating uncertainty in the phylogenetic relationships and resulting in discussion about their conservation status. For example, there is no doubt that sturgeon and paddlefish are monophyletic groups [15,18,20,21,22,23,24,25,26,27,28]. However, most molecular results have indicated that the genus *Huso* is not a monophyletic group: *H. dauricus* and *H. huso* are nested in different positions within the genus *Acipenser* [15,18,22]. Meanwhile, Findeis (1997) and Artyukhin (2010) suggested the opposite based on morphological evidence [28,29]. Moreover, *A. sinensis* and *A. dabryanus* were found by Zhang et al. (2000) to be sister species based the mitochondrial genes NADH dehydrogenase subunit 4L (ND4L) and NADH dehydrogenase subunit 4 (ND4) [20], but cytochrome b (*cytb*), 16S ribosomal DNA (rDNA), and 12S rDNA did not confirm this [25]. Furthermore, *A. oxyrinchus* and *A. sturio* were deemed related based on the similarity of eight mitochondrial sequences (12S ribosomal (rRNA), 16S rDNA, *cytb*, tRNA_Asp_, tRNA_Phe_, NADH5, and control region) by Krieger et al. (2010) [15], but this finding did not agree with that of Birstein and DeSalle (1998) [25].

As such it is difficult to address the phylogenetic relationships within the Acipenseriformes using only mitochondrial data [15]. However, many species of Acipenseriformes are polyploid because of widespread genome duplication [22], complicating the search for nuclear molecular markers. With the development of sequencing technologies and analysis methods (especially based on transcriptome and reference genome development), nuclear markers have been reported in other species [6,7,11], providing frameworks for the development of such markers in the Acipenseriformes. To date, there are only a few examples of transcriptome data, such as *A. oxyrinchus* [30], *A. sinensis* [31], *A. baerii* [32], *A. transmontanus* [33], *A. schrenckii* [34], *A. naccarii*, and *A. stellatus* [35]. Fortunately, the available genome of *Lepisosteus oculatus* [36], transcriptomes of sturgeon, and previous studies developing nuclear markers provide new foundations to analyze the phylogenetic relationships within the Acipenseriformes.

In the present study, we aimed to develop a similar approach to generate new nuclear protein-coding (NPC) markers for phylogenetic analysis using a transcriptome plus reference genome strategy. Four new transcriptomes of sturgeon and the *L. oculatus* genome were used as a database. These were analyzed by screening candidate single-copy genes to develop NPC markers and using multi-sequence alignment to find the conserved regions of exons. Two pairs of degenerate primers were designed for each marker for polymerase chain reaction (PCR) amplification. Thus, by first constructing a phylogenetic relationship and then reinvestigating the divergence times of the 16 Acipenseriformes species using the 30 newly developed NPC markers, our study provides new insight into Acipenseriformes phylogeny and improves our understanding of evolution at the nuclear gene level.

## 2. Materials and Methods

### 2.1. Datasets

The transcriptome sequencing data of seven species (*Acipenser baerii*, *A. gueldenstaedtii*, *A. naccarii*, *A. schrenckii*, *A. sinensis*, *A. stellatus*, and *A. transmontanus*) were searched and downloaded from the NCBI Sequence Read Archive (SRA) database (https://www.ncbi.nlm.nih.gov/sra). Due to the difference in data size, here we only kept the transcriptome data of four species (*A. baerii*, *A. schrenckii*, *A. sinensis*, and *A. transmontanus*) for subsequent analysis. The accession numbers and other details are presented in Table S1. The SRA files were converted into fastq files using fastq-dump within the SRA Toolkit version 2.7.0 (Bethesda, MD, USA).

### 2.2. Quality Control and De Novo Transcriptome Assembly

The fastq data were first processed using fastqc version 0.11.3 software [37] (Babraham Institute, Cambridge, UK) to view the summary quality control of the reads. Trimmomatic version 0.33 [38] (RWTH Aachen University, Aachen, Germany) was used to filter the base reads for low quality and artificial errors in the sequence ends. In this step, bases with abnormal content of approximately 8 bp according to fastqc results and those showing low quality (Q ≤ 3 base) at the start and end of the sequence were removed. The de novo assembly of clean reads was performed using Trinity version 2.0.6 software [39] (Broad Institute, Cambridge, MA, USA), with the min_kmer_cov value set to 2 and all other parameters set to default. Only contigs longer than 200 bp were used for further analysis. Then, the unigenes were extracted from the transcripts and searched against the protein data set from the *L. oculatus* genome using blastx version 2.2.31 (NCBI, Bethesda, MD, USA) with an E-value of 1 × 10^−5^. Protein data sets were extracted from the blast results based on the Perl script. The coding sequences (CDS) and protein sequences of the unigenes without blastx hits were predicted using TransDecoder version 3.0.0 (http://transdecoder.sourceforge.net/).

### 2.3. Comparison of Transcripts and Extraction of Protein Sequences

The unigene sequences of four species (*A. baerii*, *A. sinensis*, *A. schrenckii*, and *A. transmontanus*) were compared with the protein sequences from the spotted gar (*L. oculatus*) whole genome using blastx (E-value < 10^−5^). The protein sequences were then extracted according to the alignment results using the Perl script.

### 2.4. Identification of Orthologous Single-Copy Genes

We identified sturgeon orthologous genes using OrthoMCL version 2.0.9 [40] (http://www.orthomcl.org). Briefly, all protein sequences of the four sturgeons and the spotted gar were filtered according to length, with sequences >30 amino acid retained. Sequences between the start codon and the first stop codon which were less than 20% of the complete protein sequence were removed. We then used the all-vs-all BLAST method in the filtered files, and results with E-values < 10^−5^ were retained. Clustering was performed using Markov cluster algorithm (MCL) to obtain gene families. Finally, the orthologous single-copy genes (1:1:1) were extracted using the Perl script.

In addition, the spotted gar genome and the corresponding annotation files were downloaded from Ensembl (http://www.ensembl.org/index.html). We used Perl scripts to determine the corresponding genes of the single-copy orthologous sequences of the four-sturgeon species, and single-copy genes containing at least one exon of >700 bp were identified for further analysis.

### 2.5. Exon Search and Primer Design

Nested PCR and degenerate primer strategies have been widely used in developing NPC markers because they can significantly improve the success rate of PCR [5,12,41]. In order to ensure the universality of primers, we screened 193 nuclear single-copy genes, which were selected from 1850 candidate genes containing at least one exon >700 bp. First, we downloaded specific information in fasta format for the 193 candidate genes of spotted gar from Ensembl (http://asia.ensembl.org). Then, we chose 43 nuclear single-copy genes for primer design, many having only one exon. We then integrated the information for each gene in a table and performed multiple alignments of the CDS from the five species. Conserved regions containing no termination codons were selected for designing degenerate primers (Figure S1). The length of each primer was 21–26 bp, with approximately 172 primers designed for the development of nuclear gene markers.

### 2.6. Taxon Sampling, DNA Extraction, and Experimental Testing

Sixteen Acipenseriformes species were sampled in this study, consisting of four genera (Table 1). Originally, specimens were identified based on morphological characteristics [28]; but species were also identified by sequencing of mitochondrial genes [1,18,22]. All animal experiments were conducted and approved by the Animal Care and Use Committee of Southwest University (No. 2017-7). Genomic DNA was extracted from fin tissues and egg previously stored at −80 °C, using the Qiagen DNeasy Kit (Qiagen, Shanghai, China), according to manufacturer’s instructions. The 43 NPC candidate loci were amplified using nested PCR. First-round PCR reactions were conducted in 25 μL volumes containing the following: 2.5 μL 10× buffer (Mg^2+^ plus), 2.0 μL of 2.5 mM dNTPs, 1 U Taq DNA polymerase (rTaq, TaKaRa; Dalian, China), 1 μL of each first-round primer (10 μM), 1 μL genomic DNA (~100 ng/μL), and double-distilled water to yield a final volume of 25 μL. The following conditions were used for PCR amplification: initial denaturation for 4 min at 94 °C; 35 cycles of 45 s at 94 °C, 40 s at 45 °C, and 2 min at 72 °C; and a final extension of 10 min at 72 °C [12]. Negative controls (i.e., containing no DNA templates) were used in each PCR run to test for contamination and artifacts. Second-round PCR (25 μL) contained the following: 2.5 μL 10× buffer (Mg^2+^ plus), 2.0 μL of 2.5 mM dNTPs, 1 U Taq DNA polymerase (rTaq, TaKaRa; Dalian, China), 1 μL of each second-round primer (10 μM), 0.8–1.5 μL first-round PCR product without dilution, and double-distilled water to yield a final volume of 25 μL. The following conditions were used for PCR amplification: an initial denaturation for 4 min at 94 °C; 30 cycles of 45 s at 94 °C, 40 s at 50–52 °C, and 1.5 min at 72 °C; and a final extension of 10 min at 72 °C. Samples were then cooled to 4 °C. Second-round PCR products were examined via electrophoresis through 1% agarose gels. If multiple bands were present, the correct PCR product according to size was purified by cutting the band from the gel. In order to verify that markers were effective, extraction and amplification of these markers was attempted using the relevant primer pair in three species (*A. dabryanus*, *A. sinensis*, and *Polyodon spathula*). When single-band amplification, sequencing, and BLAST searching against the target gene in these three species were satisfied, the marker was tested in the remaining 13 species. An NPC candidate locus was considered a feasible marker if Sanger sequencing was successful and results were achieved in at least 13 out of the 16 species (Figure S1).

### 2.7. Sequence Assembly and Phylogenetic Analyses

Second-round PCR products were sequenced using Sanger sequencing. The forward and reverse sequences of each NPC maker were manually assembled using ContigExpress version 3.0.0 (Invitrogen; Carlsbad, CA, USA) in the Vector NIT software suite [42] and visually examined (Figure S1). The sequences were aligned using the clustalW algorithm in MEGA version 7 [43]. Ambiguously aligned regions were removed using Gblocks version 0.91b [44], with all gaps allowed (−b5 = a), utilizing the ‘codon’ model setting and setting all other values to default. We used Sequence Matrix version 1.8 [45] to concatenate successful NPC markers. The variable sites (variability) and parsimony information (PI) sites of each alignment and concatenated data were calculated by MEGA 7. The supermatrix data were divided into four parts for saturation testing by DAMBE version 5 [46]. Then, we manually defined five partitioning strategies: two partitions (1- and 2-codon positions together as a partition and one partition for the 3-codon position), three partitions (one partition for each codon position), 30 partitions (each gene as a partition), 60 partitions (one partition for 1- and 2-codon positions together and the 3-codon position as a partition across 30 genes), and 90 partitions (codon position partitioning across 30 genes). The comparisons of the five partitioning strategies and selections of corresponding nucleotide substitution models were conducted under the corrected Akaike information criterion implemented in PartitionFinder version 2 [47]. 

The supermatrix dataset was separately analyzed with both maximum likelihood (ML) and Bayesian inference (BI) methods with 60 schemes. RAxML version 8.0 [48] was used to analyze the partition ML, with the GTR + Γ + I model assigned for each partition. Branch support for the resulting phylogeny was evaluated with 500 rapid bootstrapping replicates (with −f as an option) implemented in RAxML. MrBayes version 3.2 [49] was used to analyze partition BI. All model parameters were unlinked, with one cold chain and three heated chains for the Markov chain Monte Carlo (MCMC) process, which began with random starting trees. The analysis was run for 30 million generations and sampled every 1000 generations, with the first 25% representing burn-in. Posterior probabilities were obtained from the 50% majority rule consensus tree of the remaining topologies using TreeAnnotator, and the above process was run independently twice. Chain stationarity was visualized by plotting likelihoods against the generation number using the program Tracer version 1.7 [50]. Effective sample sizes greater than 200 ensured the convergence of the results.

The construction of the species tree was completed by ASTRAL version 5.5.9 [51] based on gene trees estimated from the 30 NPC markers. This Java program estimates a species tree given a set of unrooted gene trees and support is calculated using local posterior probabilities [52]. We used 30 maximum likelihood gene trees as input trees, derived using the GTR + Γ + I model in RAxML with five hundred bootstrap replicates.

### 2.8. Hypothesis Testing

To test the statistical significance of alternative hypotheses for the Acipenseridae family within the Acipenseriformes, we compared several previous studies [15,24,28,29] with respect to the relationships among three genera within the family (i.e., *Pseudoscaphirhynchus*, *Huso*, and *Acipenser*). Topology tests were conducted using the datasets of all 16 species. The site-log-likelihood values were calculated under the GTR + Γ + I model for nucleotides using TREE-PUZZLE version 5.3 [53] (command-line option: -wsl). The obtained values were used as input for the software, CONSEL [54]. Hypotheses were statistically tested by AU [55], KH [56], and SH [57].

### 2.9. Estimating Divergence Time

In order to increase the speed of calculation, molecular dating was estimated using the relaxed-clock based program MCMCTREE version 4.9e (University College London, London, UK) [58]. In this analysis, the best tree from the hypothesis testing was used as the reference tree. All 30 NPC markers were concatenated as a single “supermatrix” (20,946 bp), and the alignment was divided into three partitions corresponding to the 1st, 2nd, and 3rd codon sites. The approximate likelihood method (usedata = 2) [58] was used for divergence time estimation. For each locus, the root age was set at “<2.0” and the model at “HKY85 + G”. The molecular clock model used independent rates (clock = 2). At present, there is only one report on the complete estimation of divergence times between species within the Acipenseriformes order [18]. Therefore, two nodes (C1 and C2) were considered as time-calibrated points with lognormal distributions and soft constraint bands (allowing violation probability of 0.025) in the present study. The C1 calibration point was estimated as the split time between sturgeon and paddlefish, based on a fossil of a Hauterivian-age polyodontid, dating from Early Cretaceous (~132–200 Mya) [17,59]. The C2 calibration point was used to estimate the node that *A. oxyrinchus* and *A. sturio* splitting with other sturgeons based on the acipenserid fossil from the Campanian age, dating from Late Cretaceous (~ 74 Mya) [60] (Table S2). We used 500,000 generations for the Markov chain Monte Carlo (MCMC) analysis, with the first 20% of all samples discarded as burn-in and samples collected every 200 generations thereafter up to 20,000. An independent rates model (clock = 2), which follows a lognormal distribution, was used for the MCMC search.

## 3. Results

### 3.1. Quality Control and Assembly

After removing artifact erroneous bases at the ends of the sequences and low-quality reads, the high-quality reads were used for further analysis. The software, Trinity [39], was used to guarantee transcripts. In the same species, all reads from different libraries or tissues were pooled. The longest isoform of a gene served as the unigene. Finally, we obtained 13,666, 125,179, 211,209, and 234,179 unigenes with an N50 of 787, 653, 654, and 704 bp from *A. sinensis*, *A. transmontanus*, *A. schrenckii*, and *A. baerii*, respectively.

### 3.2. Identification of Putative Proteins

We analyzed the possible protein sequences to identify orthologous genes for comparison of the species divergence of sequences. The unigenes of each species were used to predict CDS and protein sequences. We recovered 37,751, 36,648, 32,470, and 36,490 protein sequences (N50: 224, 216, 319, and 323 bp, respectively) from *A. sinensis*, *A. transmontanus*, *A. schrenckii*, and *A. baerii* based on the search results from blastx. The remaining unigenes were predicted using TransDecoder pipeline, obtaining 9530, 7485, 12,226, and 14,040 amino acid sequences with N50 lengths of 195, 188, 177, and 191 bp, for the four species respectively (Table S3). These two groups of protein sequences were combined and used for the identification of homologous genes. Finally, these amino acid sequences from *A. sinensis*, *A. transmontanus*, *A. schrenckii*, and *A. baerii* were used for further analysis. A total of 21,820 homologous gene families were obtained in four sturgeons and spotted gar protein sequences clustered by using OrthoMCL. Among them, 1850 single-copy orthologous genes were identified, including spotted gar genes. In addition, 193 nuclear single-copy genes that contained at least one exon of >700 bp were selected from the spotted gar genome.

### 3.3. Characteristics of NPC Markers

According to the abovementioned screening criteria, we successfully found 30 new NPC markers for Acipenseriformes, which were amplified in at least 14 species. Fragments ranged from 467 bp to 993 bp, with an average length of 698 bp. In a total of 480 PCR reactions (30 loci × 16 species), 476 (99.1%) were successfully amplified, with 469 (97.7%) producing high-quality bands used for Sanger sequencing (Figure S2). The PCR primer information of the 30 NPC markers, including their genome locations, is listed in Table S4 and their GenBank accession numbers are shown in Table S5. Their variability ranged from 4% to 16%, with an average of 9%, and their parsimony-informative sites ranged from 2% to 16%, with an average of 4% (Table S6). Twenty-seven NPC markers were amplified in all 16 species. The NPC marker ‘*fam43a*’ could not be amplified from *H. dauricus* or *Pseudoscaphirhynchus kaufmanni*, and ‘ENSLOCG00000018097’ and ‘*cebpa*’ could not be amplified from *P. kaufmanni* (Figure S2).

### 3.4. Reconstructing Phylogenetic Relationships in the Acipenseriformes

The supermatrix combined 30 NPC markers based on 20,946 bp. The results of the saturation tests showed that the values of the saturation index (*Iss*) for the first codon, second codon, third codon, first and second codons, and all three sites of protein-coding regions were significantly smaller than the critical values, *Iss.cSym* or *Iss.cAsym* (Table S7). 

**Figure 1:**
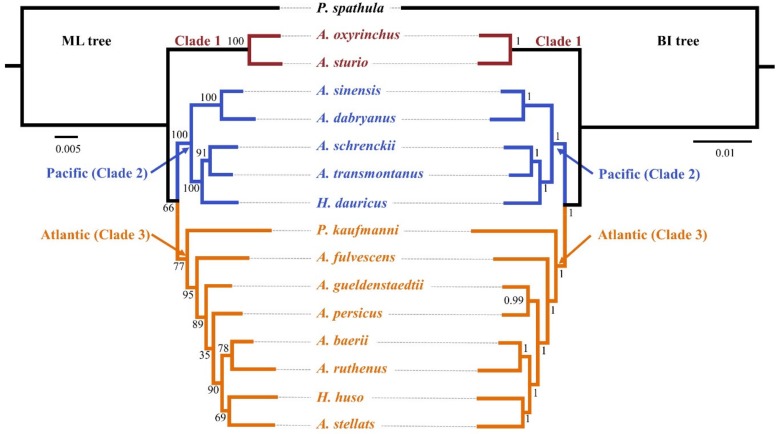
Phylogenetic relationships of Acipenseriformes inferred from 30 nuclear protein-coding (NPC) markers. Left: maximum likelihood (ML) tree; right: Bayesian inference (BI) tree; bootstrap support and Bayesian posterior probability are shown on the nodes.

**Figure 2:**
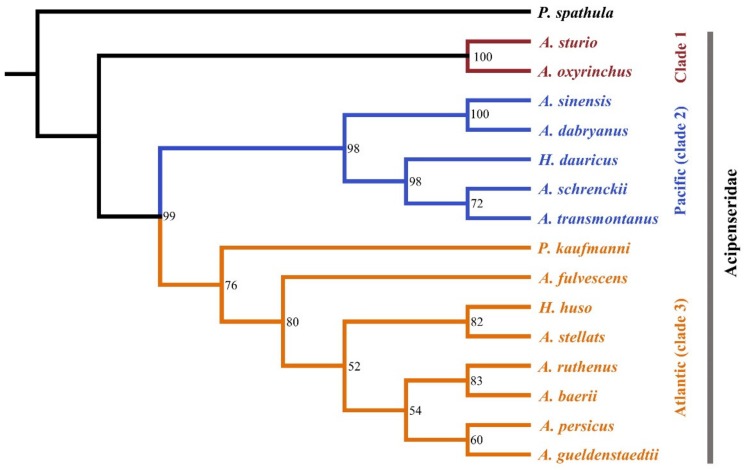
Species tree estimation of Acipenseriformes based on 30 NPC markers using the method of accurate species tree algorithm (ASTRAL). Bootstrap support is shown on the nodes.

The results of PartitionFinder 2 supported the 60 schemes as the best-fitting partitioning schemes using both ML and BI analyses. Both the ML tree and BI tree produced highly similar topologies. Ten out of thirteen internal nodes produced bootstrap support of >75% in the ML tree and all internal nodes had posterior probabilities >0.95 in the BI tree (Figure 1). The species tree produced similar topologies with ML and BI trees in most lineages, and most internal nodes had high branch support (Figure 2). The gene and species trees can divide the Acipenseridae into three clades: clade 1, containing *A. sturio* and *A. oxyrinchus* as sister species; clade 2, containing the Pacific species *A. dabryanus*, *A. sinensis*, *A. schrenckii*, *A. transmontanus*, and *H. dauricus*; and clade 3, containing the Atlantic sturgeons *A. baerii*, *A. fulvescens*, *A. gueldenstaedtii*, *A. persicus*, *A. ruthenus*, *A. stellatus*, *H. huso*, and *P. kaufmanni*.

### 3.5. Hypothesis Testing

The topology test strongly supported our gene tree (Table 2). The genus *Huso* was not found to be a monophyletic group, where *H. dauricus* and *H. huso* were dispersed and nested within the genus *Acipenser*. *P. kaufmanni* was also nested in the genus *Acipenser*. The topologies that rejected the hypothesis were (*Pseudoscaphirhynchus* + (*Huso* + *Acipenser*)) [28] and (*Huso* + (*Pseudoscaphirhynchus* + *Acipenser*)) [29]; both were based on morphological traits.

### 3.6. Divergence Times

**Figure 3:**
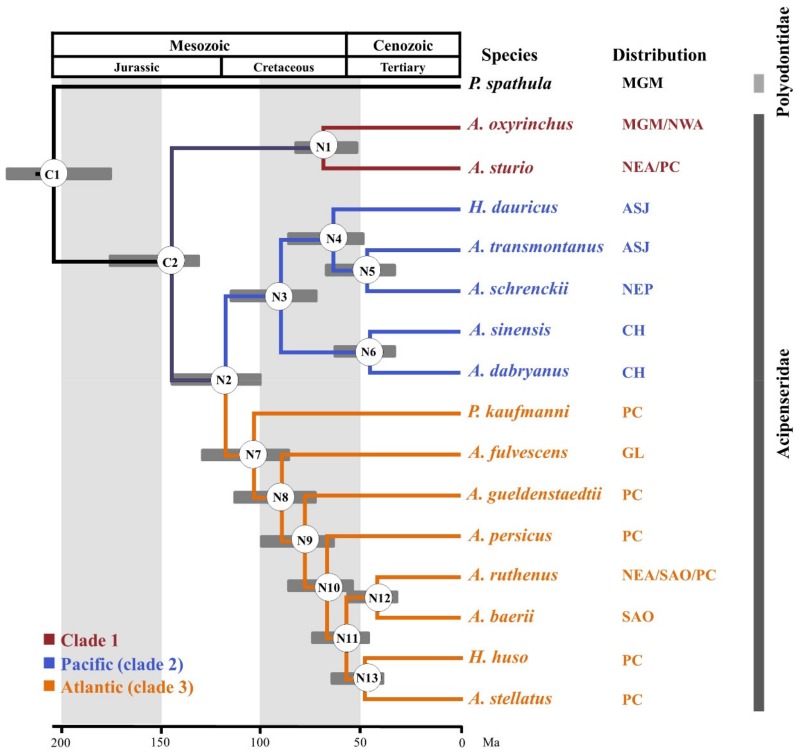
Timetree of 16 Acipenseriformes species. The node number represents the corresponding number. C1 and C2 are calibration nodes. N1–N13 indicate the nodes of interest for which estimated dates are presented in Table S8. CH: China; ASJ: Amur River, Sea of Okhotsk, Sea of Japan; NEP: Northeast Pacific.

The estimated divergence times of the 16 species of Acipenseriformes are shown in Figure 3. Our results suggest that the estimated divergence of sturgeon and paddlefish occurred at 204.1 Mya, with a 95% credibility interval of 180.3–233.6 Mya. Our times are not only earlier than the 141.4 Mya and 184.4 Mya [18] calculated based on partial mitochondrial genome sequences and *cytb* data, respectively, but are also earlier than the results of 114.1 Mya for data set 1 and 145.2 Mya for data set 2 obtained by Inoue et al. (2005) [61]. The divergence time of *A. sturio*-*A. oxyrinchus* clade and the other sturgeon species was estimated about 144.9 Mya, with a 95% credibility interval of 129.9–175.3 Mya. The Pacific-Atlantic split in this species occurred at 117.6 Mya (95% credibility interval of 98.9–144.8 Mya). *P. kaufmanni* separated at 103.3 Mya (95% credibility interval of 85.3–128.0 Mya). In addition, the *A. sinensis* and *A. dabryanus* divergence time dated back to 44.7 Mya, with a 95% credibility interval of 31.4–61.8 Mya.

## 4. Discussion

Polyploidization is a common phenomenon in animals and plants [62], which unfortunately causes problems for the use of nuclear markers in phylogenetic analyses. These problems make conventional methods of nuclear marker development very inefficient. On the contrary, searching conservative sequences based on multi-sequence alignment provides a new method to develop large scale nuclear markers [5,7,9,63]. Many Acipenseriformes species are polyploid and lack reference genome sequence data; as a result, there has been strong dependence on mitochondrial data of the phylogenetic relationships among species, with nuclear data rarely used. Nowadays, most Acipenseriformes are rare and often close to extinction, making sampling of wild specimens very difficult.

Twenty to fifty loci are often sufficient to answer phylogenetic questions [64,65]. Therefore, we used a strategy of multi-sequence alignment of transcriptomes combined with spotted gar genomic data to develop single-copy NPC markers to construct the phylogenetics of Acipenseriformes. The success rate of PCR amplification is an important parameter [41]. Our success rate was 99.1%, representing 30 NPC markers (100% for 27 markers) in 16 species of Acipenseriformes (Figure S2); the combination of low degenerate primers and nested PCR was important for this high rate of success. Phylogenetic analysis showed that the gene and species trees have highly similar topologies and most internal nodes are highly supported, showing that our NPC markers are reliable. This strategy for developing NPC markers can be used for the development of molecular markers in other polyploid groups that also lack reference genomes but have sufficient transcriptome data. Notably, the set of 30 NPC markers can be used for the remaining 11 sturgeon species.

The results from the nuclear gene trees and species tree strongly support the classification of Acipenseriformes into two families, Acipenseridae and Polyodontidae, which is consistent with previous morphological and molecular studies [15,18,21,22,23,24,25,27,28,51,66]. The monophyly of sturgeon and paddlefish is not disputed. Nonetheless, the phylogenetic relationships of the four genera in the family Acipenseridae have been controversial for a long time. Our results suggest the existence of three clades, where clade 1 is represented by *A. sturio* and *A. oxyrinchus* as sister species with a unified basal lineage. All other species of *Acipenser*, *Huso*, and *Pseudoscaphirhynchus* can be divided into Pacific species (clade 2) and Atlantic species (clade 3) [15,18]. Our data support the Pacific clade being a monophyletic group, in agreement with previous molecular studies [15,18,22,26]. However, the internal phylogenetic relationships are different from those of previous studies. For example, *A. dabryanus* and *A. sinensis* have been found to be a sister group with *A. schrenckii* and *A. transmontanus* based on many mitochondrial DNA data, but nuclear markers suggest that *A. schrenckii* and *A. transmontanus* should be a sister group with *H. dauricus*. Presently, *A. dabryanus* and *A. sinensis* are found in China; *H. dauricus* and *A. schrenckii* are found in the Amur River, Sea of Okhotsk, and Sea of Japan; and *A. transmontanus* is found in the North Eastern Pacific (Table 1, Figure 3). Therefore, our results are consistent with geographical distribution of species. Currently, the species composition of the Atlantic clade is complex and contains at least three genera, including *Acipenser*, *Huso*, and *Pseudoscaphirhynchus*. The genus *Huso* is not a monophyletic group, with *H. dauricus* and *H. huso* dispersed and nested within the genus *Acipenser*, consistent with previous molecular studies [15,18,22]. The genus *Pseudoscaphirhynchus* is also nested in the genus *Acipenser*. Previous studies have indicated that *P. kaufmanni* is closely related with *A. stellatus* within the Atlantic clade [15,24]. However, our data do strongly support that *P. kaufmanni* is located basal in the Atlantic clade, which has not been noted previously.

Prior to this study, there was only one complete report on the divergence time of 24 species of Acipenseriformes; therefore, we choose *cytb* results for comparison [18]. Considering the slower saturation of nuclear exon data, time estimates based on nuclear exons would seem to be more reliable [67]. Notably, our results showed that the nuclear gene data set was narrower and more accurate in its credibility intervals. We estimated the divergence time for sturgeon and paddlefish at 204.1 Mya, similar to the oldest Acipenseriformes fossil record (ca. 200 Mya) [17,59]. The Ponto-Caspian region has been very unstable over the last 150 Mya [68], which may have led to the diversification of the Acipenseridae [26]. Based on our dating results, the divergence time of Acipenseridae was estimated to be 144.9 Mya (Table S8), more recently than the 171.6 Mya estimated previously [18], but close to 150 Mya, supporting the hypothesis of Bemis and Kynard (1997) [26]. Interestingly, we determined that the Pacific-Atlantic split occurred at around 117.6 Mya, around the time that the Tethys Sea separated into two oceans (120 Mya) [68]. We suggest that the continued shrinkage of the Tethys Sea greatly changed the habitat, resulting in a significant diversification of the sturgeon species. Additionally, the divergence time of the genus *Pseudoscaphirhynchus* was estimated to be 103.3 Mya, less recently than estimated by all previous studies. However, due to the incomplete sampling of species, the actual divergence time may be a little earlier than our results indicate. 

## 5. Conclusions

The transcriptome plus reference genome data mining strategy utilized here to identify suitable nuclear protein-coding loci for novel marker development has been proven successful in Acipenseriformes. The newly developed 30 NPC markers had a high rate of experimental success (~99.1%), with excellent performance of the concatenated dataset describing phylogenetic relationships. This dataset and markers were useful in reconstructing the relationships within the Acipenseriformes order, yielding the novel findings that *P. kaufmanni* is identified located in the base position of the Atlantic clade of species. As more sequences become available in the future, further studies with more comprehensive taxon sampling are essential to fully resolve the evolutionary history of Acipenseriformes. Additionally, our marker set offers a great opportunity for species identification including artificial produced hybrids. Trade control is essential for protection of last remaining wild stocks.

## Figures and Tables

**Table 1:** Sample collection list of all species used in this study.

Species	Common Name	Collection Locality	Distribution ^1^
*Acipenser*			
*A. baerii*	Siberian sturgeon	Germany	SAO
*A. dabryanus*	Yangtze sturgeon	Yibin, Sichuang, China	CH
*A. fulvescens*	Lake sturgeon	Wolfgangsee, Wisconsin, America	GL
*A. gueldenstaedtii*	Danube sturgeon	Germany	PC
*A. oxyrinchus*	Atlantic sturgeon	Canada	MGM/NWA
*A. persicus*	Persian sturgeon	Iran	PC
*A. ruthenus*	Sterlet sturgeon	The Danube, Germany	NEA/SAO/PC
*A. schrenckii*	Amur sturgeon	Wanzhou, Chongqing, China	ASJ
*A. sinensis*	Chinese sturgeon	Yangtze river fisheries research institute, Hubei, China	CH
*A. stellatus*	Starry sturgeon	The Danube, Romania	PC
*A. sturio*	Sturgeon	Gironde River, France	NEA/PC
*A. transmontanus*	White sturgeon	Amur river, China	NEP
*Huso*			
*H. dauricus*	Kaluga	Amur river, China	ASJ
*H. huso*	Beluga	The Danube, Romania	PC
*Pseudoscaphirhynchus*			
*P. kaufmanni*	Amu Darya sturgeon	Amu Darya, Turkmenistan	PC
*Polyodon*			
*P. spathula*	Paddlefish	Wanzhou, Chongqing, China	MGM

^1^ NEP: North Eastern Pacific; GL: Great Lakes, Hudson Bay & St. Lawrence R., NWA: North Western Atlantic; MGM: Mississippi R. & Gulf of Mexico; NEA: Northeastern Atlantic, including White, Baltic & North seas; PC: Ponto-Caspian Region, including Mediterranean, Aegean, Black, Caspian & Aral seas; SAO: Siberia & Arctic Ocean; ASJ: Amur R., Sea of Okhotsk & Sea of Japan; CH: China (Bemis and Kynard, 1997).

**Table 2:** Tree topology testing.

Rank	Topology Tested	log L	AU	KH	SH
1	Best tree (ML tree)	−47,716.74	1.000	1.000	1.000
2	(((((ASTE,PK),AR)),HH),(((AT,ASC),(ASI,AD)),HD) ^1^	−47,944.33	6 × 10^−^^8^	0	0.011
3	((ASTE,PK),((AR,HH),HD),AF) ^2^	−48,422.00	2 × 10^−^^25^	0	0
4	((*Acipenser*, PK),(HH,HD)) ^3^	−48,869.58	0.008	0	0
5	(((((HH,HD),(AR,ASC)),ASTE),(ASTU,AO)),PK) ^4^	−48,915.92	2 × 10^−^^62^	0	0

^1^ Krieger et al., 2010 [15]; ^2^ Birstein et al., 2002 [24]; ^3^ Findeis, 1997 [29]; ^4^ Artyukhin, 2010 [28]. AD = *Acipenser dabryanus*, AO = *Acipenser oxyrinchus*, AR = *Acipenser ruthenus*, ASC = *Acipenser schrenckii*, ASTE = *Acipenser stellatus*, ASI = *Acipenser sinensis*, ASTU = *Acipenser sturio*, AT = *Acipenser transmontanus*, HD = *Huso dauricus*, HH = *Huso huso*, PK = *Pseudoscaphirhynchus kaufmanni.*

## Supplementary Materials

The following are available online at https://www.mdpi.com/2073-4425/10/1/38/s1, Figure S1: Flowchart for bioinformatics screening and experimental design, Figure S2: PCR amplification rendering of the 30 NPC markers in 16 Acipenseriformes species, Table S1: Transcriptome information of four sturgeons, Table S2: Maximum (U) and minimum (L) constrains (Mya) and calibration information for nodes in Figure 1 in the molecular dating analysis, Table S3: Number of predicted protein sequences (N) according to detected unigenes, Table S4: Summary information of 30 NPC markers, Table S5: List of GenBank accession numbers for the markers obtained in the present study, Table S6: Summary information of 30 NPC markers in 16 species of Acipenseriformes, Table S7: Saturation test performed by using DAMBE, Table S8: Detailed results of Bayesian molecular dating using MCMCTREE, and a comparison among shared nodes in previous study.
