# Supplementary figures and images for: Highly Resolved Phylogenetic Relationships within Order Acipenseriformes According to Novel Nuclear Markers
Source: Genes (Basel). 2019 Jan 10;10(1):38. doi: 10.3390/genes10010038 (PMC6356338; doi:10.3390/genes10010038)

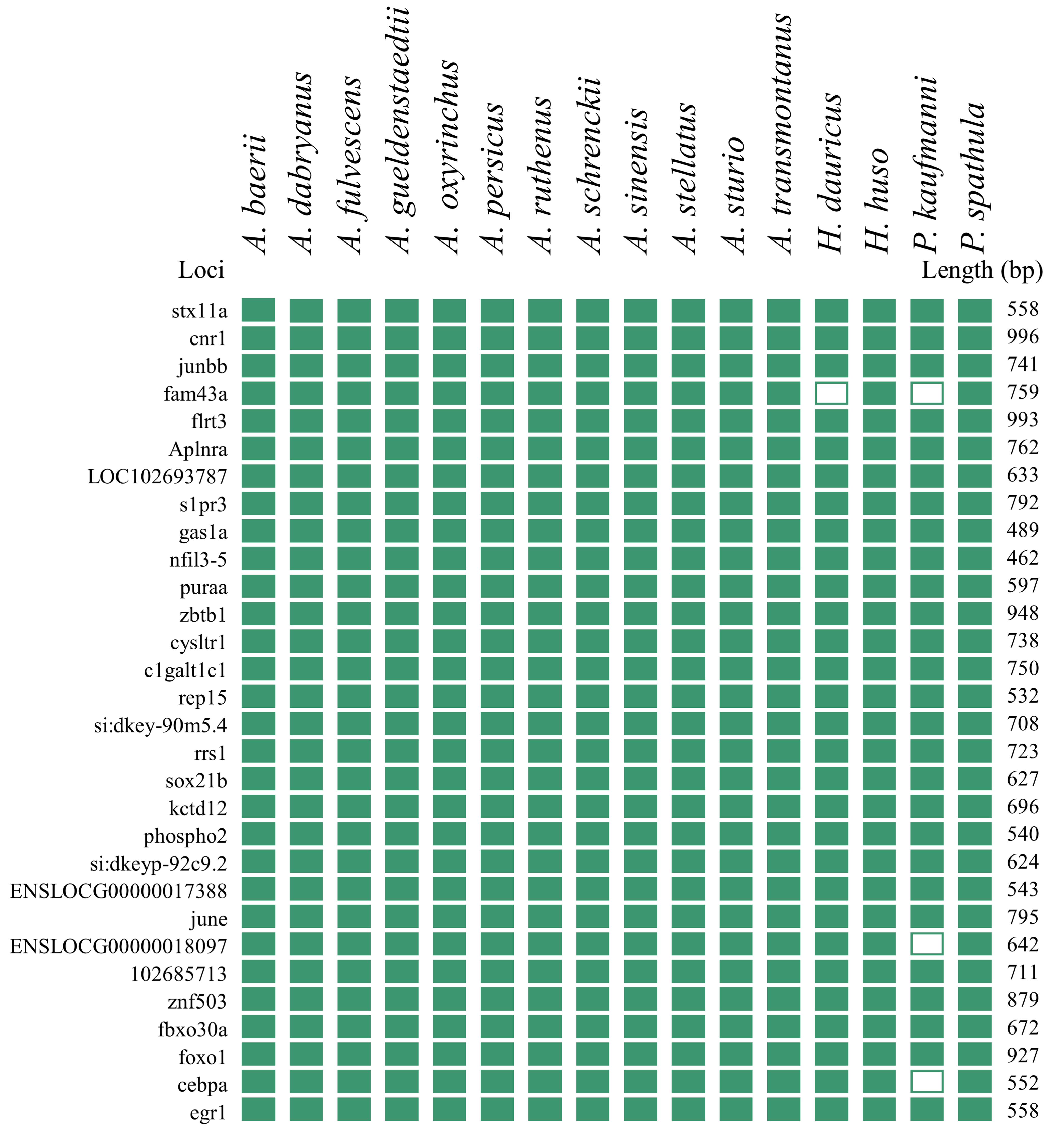

Supplement: Supplementary file 1 [file genes-10-00038-s001.zip › Neuer Ordner mit Objekten/Figure S2.tif]

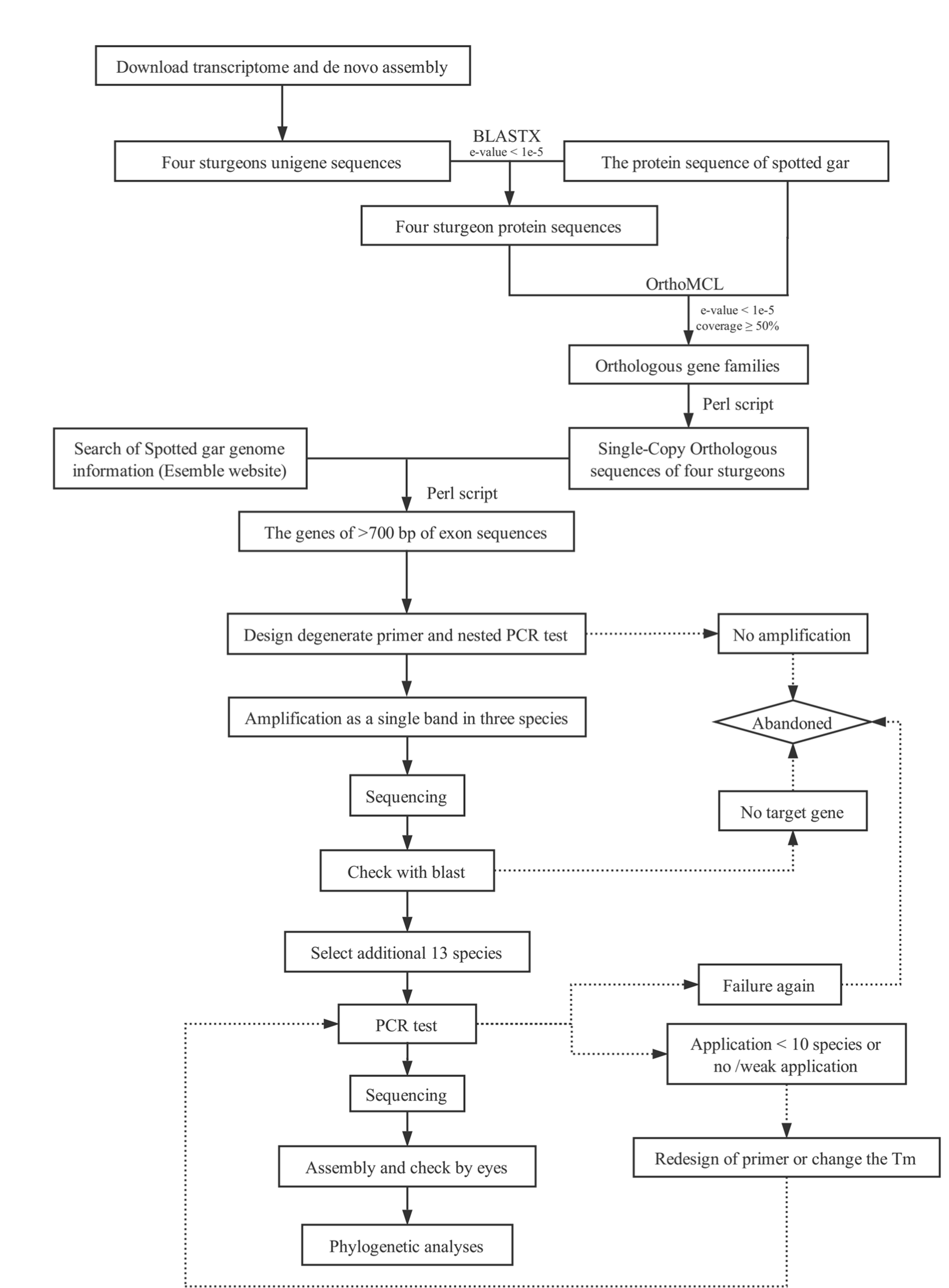

Supplement: Supplementary file 1 [file genes-10-00038-s001.zip › Neuer Ordner mit Objekten/Figure S1.tif]
